# Inhibition of *PKCθ* Abrogates CD8^+^ T Cell-Mediated Neurotoxicity in Murine Cerebral Malaria

**DOI:** 10.3390/biomedicines13112582

**Published:** 2025-10-22

**Authors:** Karin Albrecht-Schgör, Victoria E. Stefan, Martina Steinlechner, Dominik Humer, Kerstin Siegmund, Sebastian Peer, Thomas Gruber, Maja Überegger, Stephanie zur Nedden, Gabriele Baier-Bitterlich, Peter Lackner, Erich Schmutzhard, Nikolaus Thuille, Victoria Klepsch, Gottfried Baier

**Affiliations:** 1 Institute for Cell Genetics, Medical University of Innsbruck, 6020 Innsbruck, Austria; karin.albrechtschgoer@gmail.com (K.A.-S.); martina.steinlechner@student.i-med.ac.at (M.S.); dominik.humer@i-med.ac.at (D.H.); kerstin.siegmund@i-med.ac.at (K.S.); thomas.gruber@i-med.ac.at (T.G.); nikolaus.thuille@i-med.ac.at (N.T.); gottfried.baier@i-med.ac.at (G.B.); 2 Research Program for Receptor Biochemistry and Tumor Metabolism, Department of Pediatrics, University Hospital of the Paracelsus Medical University Salzburg, 5020 Salzburg, Austria; v.stefan@salk.at; 3 Department of Biosciences and Medical Biology, University of Salzburg, 5020 Salzburg, Austria; 4 Internal Medicine V (Hematology and Oncology), Medical University of Innsbruck, 6020 Innsbruck, Austria; sebastian.peer@i-med.ac.at; 5 Institute for Neurobiochemistry, Medical University of Innsbruck, 6020 Innsbruck, Austriastephanie.zur-nedden@i-med.ac.at (S.z.N.); gabriele.baier-bitterlich@i-med.ac.at (G.B.-B.); 6 Institute of Physiology, Medical University of Innsbruck, 6020 Innsbruck, Austria; 7 Vienna Healthcare Group, Department of Neurology, Klinik Floridsdorf, 1010 Vienna, Austria; peter.lackner@gesundheitsverbund.at; 8 Karl-Landsteiner-Institute for Clinical Research in Acute Neurology, 1010 Vienna, Austria; 9 Department of Neurology, Medical University of Innsbruck, 6020 Innsbruck, Austria; erich.schmutzhard@i-med.ac.at

**Keywords:** cerebral malaria (CM), experimental cerebral malaria (ECM), CD8^+^ T cells, protein kinase C-θ (PKCθ), neurovascular damage, immune-mediated brain injury, host-targeted candidate therapy, drug repurposing

## Abstract

**Background**: Cerebral malaria (CM) is a severe and often fatal complication of *Plasmodium falciparum* infection that causes devastating brain injury largely through immune-mediated mechanisms. Pathogenic brain-infiltrating CD8^+^ T cells are key drivers of CM pathology, yet the intracellular signals enabling their harmful autoimmune-like activity remain poorly defined. Here, we identify protein kinase C θ (PKCθ), a central antigen receptor-signalling mediator, as a critical contributor to experimental cerebral malaria (ECM). **Methods/Results:** Using a PKCθ null allele mouse strain on a C57BL/6N background, we demonstrate that PKCθ deficiency significantly improves survival in *Plasmodium berghei* ANKA (*PbA*)-infected mice without altering parasite burdens in the blood or brain. Mechanistically, loss of PKCθ skews T cell differentiation towards central memory (Tcm) rather than effector memory (Tem) phenotypes, thereby reducing effector differentiation and sequestration of CD8^+^ T cells in the cerebral microvasculature. This prevents extensive neurovascular damage, preserves neural tissue integrity, and alleviates neurological signs and symptoms. Our findings provide genetic evidence that PKCθ drives CD8^+^ T cell-mediated brain injury in ECM. **Conclusions:** These results underscore the potential for repurposing clinically PKCθ inhibitors as host-targeted interventions to protect against cerebral injury and improve outcomes in patients with CM.

## 1. Introduction

Cerebral malaria (CM) is a severe and frequently fatal neurological complication of *Plasmodium falciparium* malaria, which disproportionately affects children under five in sub-Saharan Africa [1]. CM is responsible for around 400,000 deaths among African children each year, accounting for over 90% of malaria-related deaths [2]. According to the 2024 WHO World Malaria Report, an estimated 597,000 global malaria deaths occurred in 2023, predominantly in sub-Saharan Africa, where CM remains a leading cause of fatal malaria complications [3]. Even with improved therapies, 15–20% of CM cases remain fatal, underscoring the need for new treatment approaches.

The pathogenesis of CM involves complex immune–vascular interactions [4]. Infected red blood cells (iRBCs) adhere to brain endothelial cells (ECs) to evade splenic clearance, via interactions between the parasite protein PfEMP-1 and host molecules, including ICAM-1, VCAM-1, CD36, and EPCR [5]. Cytokine-mediated endothelial activation amplifies inflammation and enables antigen cross-presentation via MHC-I. Activated ECs secrete CXCL-10, recruiting cytotoxic CD8^+^ T cells, which induce endothelial apoptosis through interferon-gamma (IFN-γ) and Granzyme B (GrzB) [6]. This immune cascade can disrupt the blood–brain barrier (BBB), cause hemorrhages and brain swelling, and lead to multi-organ failure including respiratory arrest [7]. CM is therefore, at least in part, an autoimmune-like disease driven by immune cell trafficking and effector functions.

Experimental cerebral malaria (ECM), caused by *Plasmodium berghei ANKA (PbA)* infection in mice, is the main preclinical model used to investigate these mechanisms [8,9]. The severity of the neurological symptoms is standardized using Innsbruck Cerebral Malaria Score (ICMS) [10]. Early studies using germline knockout of protein kinase C-theta (PKCθ)—a serine/threonine kinase central to T cell activation—suggested that PKCθ deficiency delays disease progression and improves survival. Impaired expression of adhesion molecules (such as LFA-1) has been suggested to be responsible for less brain sequestration of CD4^+^ and CD8^+^ T cells [11,12].

There are two distinct *PKCθ*-knockout strains, the ‘Littman’ and ‘Baier’ strains, which differ in their targeting strategies and T cell phenotypes [13]. The Littman strain, which has been used in previous ECM studies, retains the coding sequence for a truncated protein consisting of 365 amino acids that may act as a dominant-negative or otherwise interfere non-specifically with cellular signaling [11,12]. In contrast, the Baier strain, used in the present study, was generated by deleting exons 3 and 4 using Cre/loxP technology. This produces a true null allele expressing only a nine-amino acid peptide, ensuring that no functional or interfering PKCθ protein fragment is produced, and thus provides a more definitive model for analyzing PKCθ’s physiological roles. Using the Baier PKCθ null strain, we validated that PKCθ deficiency significantly improves the survival of *PbA*-infected mice. Mechanistically, loss of PKCθ significantly protects against fatal ECM by shifting CD8^+^ T cells towards a less pathogenic central memory phenotype, thereby reducing their effector differentiation within the cerebral microvasculature, preventing endothelial injury, preserving neural tissue, and alleviating neurological signs and symptoms. These findings of robust neuroprotection provide genetic evidence that PKCθ drives CD8^+^ T cell-mediated neurovascular damage in ECM and highlight the translational potential of repurposing clinically approved PKCθ inhibitors as host-targeted therapies to mitigate brain injury in CM. Although EXS4318 is not yet approved for clinical use, it is a highly selective and promising PKCθ inhibitor that had progressed to clinical trials by September 2025.

## 2. Materials and Methods

**Mice**: C57BL/6N and *PKCθ^−/−^* [13] mice were housed and bred in the “Zentrale Versuchstieranlage” (ZVTA) in Innsbruck under specific pathogen-free (SPF) conditions. In this facility, the animals live at room temperature and with a 12 h light-dark cycle, while food (pellets) and water are provided ad libitum. As soon as pups could be weaned or until they reached the appropriate age for the *PbA* infection (6–8 weeks), the animals were transferred to the animal facility for infectious diseases, where they were housed in individually ventilated cages (IVC). If not stated differently, male mice were chosen randomly from litters for most experiments, except for neurological score and survival, where male and female mice were used at the beginning of the project. The experiments were in accordance with ethical and legal guidelines. The Austrian Federal Ministry of Education, Science and Research approved animal experiments at the Medical University of Innsbruck (BMFWF-66.011/0102-WF/V/3b/2017 and 2022-0.573.913).

**Parasites and infections**: ECM-causing *PbA* parasites were kindly provided by Prof. Dr. Peter Lackner. *PbA* expressing GFP constitutively during the whole life cycle of the parasite (GFPcon 259cl2) was ordered from BEI Resources (ATCC, Manassas, VA, USA). The parasite was either cryopreserved in liquid nitrogen or continuously passaged from mouse to mouse. In this regard, mice that were older than 8 weeks and therefore less likely to develop CM were used as transfer mice. Their blood was collected from the heart approximately on day 4 post infection (p.i.) in 500 I.U. heparin (Gilvasan, Vienna, Austria) and was transferred to the next mouse. 7–8-week-old male and female C57BL/6N and *PKC-θ*-KO mice were infected intravenously (i.v.) with 7.5 × 10^4^ iRBCs in 50 µL saline [14]. Parasitemia, assessed by Giemsa-stained blood smears, was monitored daily.

**Figure A1:**
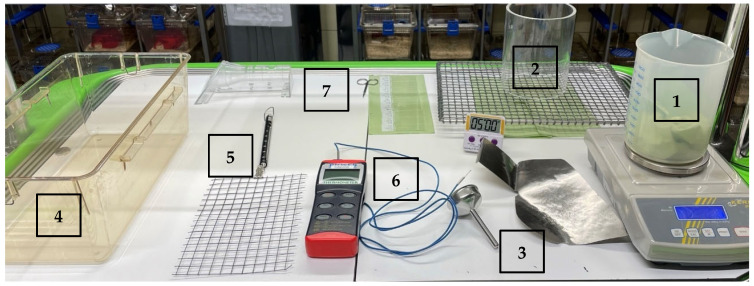
Setup to assess ECM disease progression according to ICMS including the evaluation of (1) body weight, (2) body position and spontaneous activity in a transparent cylinder and on an elevated grid, (3) the response to a sudden loud noise, (4) the gait in an empty cage and touch escape, (5) grip strength, (6) body temperature and (7) parasitemia via blood smear, as well as (not depicted) evaluating the condition of the fur (piloerection), body/abdominal/limb tone, irritability during restraint and vocalization throughout the entire course.

**Assessment of disease advancement**: The Innsbruck Cerebral Malaria Score (ICMS) is an adaption by Lackner et al. [10] of the SHIRPA score by Rogers and colleagues [14]. The ICMS is designed to detect neurological deficits which develop during ECM and includes 10 neurobehavioral parameters, such as body position, spontaneous activity, piloerection, grip strength, gait, straddle response, touch escape, irritability, vocalization, and abdominal/limb tone. The scoresheet and setup of the testing area have been added to the Appendix A (Table A1, Figure A1). During the scoring procedure, a maximum of 18 points can be reached by a healthy mouse. Down to a score of 15 points, behaviour is also considered to be within the normal range. However, a mouse manifesting mild cerebral malaria (CM I) scores 14 to 12 points. CM stage II (medium symptoms) is defined with 11 to 8 points, while a score below 8 points indicates severe cerebral malaria (CM III). Rectal body temperature and weight were monitored in addition. Male and female mice were scored for neurological symptoms and differences between the sexes. Mice were euthanized if ICMS dropped below 8 points, body weight loss exceeded 20%, temperature fell below 35 °C, or parasitemia surpassed 40%.

**Parasitemia**: To assess the level of *PbA*-infected red blood cells, blood was collected from the tail tip and used to generate a thin blood smear on a glass slide. The samples were fixed in methanol (≥99.5%, Fisher Scientific, Pittsburgh, PA, USA) for 7 min, stained with Giemsa (Sigma-Aldrich, Wien, Austria) for 2 min, and then rinsed with deionized water. Parasitemia was calculated according to the following equation:

$$\frac{number\;of\;iRBC}{number\;of\;total\;erythrocytes}=parasitemia$$

**Blood–Brain Barrier and brain swelling**: BBB integrity was tested by intravenous injection of a 2% Evans blue/PBS (*w*/*v*) solution on day 6 post-infection, as described elsewhere [15]. After 1.5 h post-injection, mice were terminally anaesthetized with 100–150 µL of ketamine (100 mg/mL)/xylazine (20 mg/mL) (Livisto)/NaCl (1:1:2) (i.p.) and perfused through the left ventricle with 0.9% cold saline for 5 min. Isolated brains were disintegrated in 2% formamide, and supernatants were analyzed spectrophotometrically at 620 nm after 48 h of incubation at 37 °C and related to a standard curve. To assess brain swelling secondary to the disintegration of the BBB, mouse brains were removed on day 6 post-infection and dried at 150 °C overnight. The drying loss was determined using an analytical balance (detailed description elsewhere [14]).

**Immunohistochemistry and immunofluorescence**: For histological investigation of brains, terminally anaesthetized mice were perfused through the left ventricle with cold saline for 5 min followed by 5 min of perfusion with 4% paraformaldehyde (PFA)/PBS (*w*/*v*). All perfusions were performed without a pump, merely driven by gravitation to avoid vascular damage. Excised brains were kept in 4% PFA overnight at 4 °C and for an additional 24 h in 30% sucrose before freezing and sectioning at 20 µm. ImageJ software (version 1.54) was used to determine the number and area of brain lesions after Hematoxylin-Eosin (H&E) staining. Brain sequestered iRBCs were identified with a GFP-expressing *PbA*. On day 6 post-infection, brains were perfused as mentioned above, and brain sections were incubated with primary antibodies against CD8 (1:500, catalogue number: PA5-81344, ThermoFisher, Waltham, MA, USA) and CD31 (1:50, clone: MEC 13.3, catalogue number: 550274, BD Biosciences, Franklin Lakes, NJ, USA). For confocal fluorescence microscopy, secondary antibodies AF647 (1:1000, catalogue number: A-21244, Invitrogen, Waltham, MA, USA) for CD8 and AF568 (1:1000, catalogue number: A-11077, Invitrogen) for CD31 were applied. Hoechst (1:1000) was used for the visualization of cell nuclei. Images were acquired using the LSM 700 Axio Observer Z1m confocal microscope, and Zen LSM software (Zeiss, Oberkochen, Germany, version 2.0) was employed for image processing.

**Isolation of brain-sequestered lymphocytes**: Terminally anaesthetized mice (day 6 post-infection) were perfused through the left ventricle with cold saline for 5 min without a pump, merely driven by gravitation. Excised brains of WT and *PKCθ*-KO mice were cut into small pieces and digested in a mix of Collagenase D (2.5 mg/mL, catalogue number: 11088858001, Roche, Basel, Switzerland) and DNase I (1 mg/mL catalogue number: 11284932001, Roche) in PBS. After 30 min incubation at 37 °C, 0.01 M EDTA (ThermoFisher, Waltham, MA, USA) was added for 5 min to prevent aggregates of T cells and dendritic cells. Digested brains were pushed through a 100 µm cell strainer and rinsed with 7 mL RPMI (PAN Biotech, Aidenbach, Germany). Percoll (catalogue number: GE17-0891-01, Merck, Darmstadt, Germany) density gradient centrifugation was performed to isolate lymphocytes from brain suspensions. Therefore, 2 mL of 70% Percoll (prepared in HBSS (Gibco, Billings, MT, USA) were carefully overlayed with 10 mL of a 30% Percoll solution containing the disintegrated brain cells (in RPMI) in a 15 mL falcon. Samples were centrifuged with 500× *g*, at 20 °C, for 30 min (medium acceleration, no brake). 3 mL of the interphase (lymphocytes) were collected and washed with 10 mL cold RPMI. The resulting cells were stained for flow cytometry.

**Flow cytometry**: Lymphocytes were isolated from the brain on day 6 post-infection by density centrifugation as described above. Prior to the perfusion of mice, spleens were harvested and mashed through a 100 µm cell strainer. Splenocytes were depleted of erythrocytes using the mouse erythrocyte lysing kit (R&D, WL2000). Thereafter, 0.01 M EDTA was added for 5 min to prevent cell aggregates. The resulting cells were filtered through a 40 µm cell strainer. Single-cell suspensions from spleens and brains were resuspended in 200 µL culture medium (RPMI from PAN Biotech, supplemented with 1% penicillin/streptavidin, 10% FCS, and 1% L-glutamine) and transferred to a 96-well flat-bottom plate (1 × 10^6^ cells/well), which has been coated with 5 µg/mL anti-CD3 (clone: 2C11), for overnight recall stimulation. The next day, cytokine release from the cells was inhibited by adding GolgiPlugTM (1 mg/mL, catalogue number: 555029, BD Biosciences, Franklin Lakes, NJ, USA) and GolgiStopTM (0.8 mg/mL, catalogue number: 554724, BD Biosciences, Franklin Lakes, NJ, USA) for 4 h. After washing with HBSS, cells were stained with fixable viability dye eFluor780 (1:1000 in HBSS, catalogue number: 65-0865-14, eBioscience, San Diego, CA, USA). PBS containing 2% fetal calf serum (Sigma-Aldrich) was used to remove excess stains. Fc receptors were blocked (catalogue number: 553142, BD Biosciences) to prevent nonspecific antibody binding before staining with respective surface antibodies for 30 min at 4 °C. After surface staining, cells were fixed and permeabilized using a fixation/permeabilization kit (catalogue number: 421002, Biolegend, San Diego, CA, USA), and intracellular staining was performed for 30 min at 4 °C. Cells were measured using the BD FACS CantoII^TM^ (BD Biosciences, Franklin Lakes, NJ, USA), and data were analyzed with FlowJo software (version 10). The following antibodies were used for flow cytometry: CD45 FITC (1:400, clone 30-F11, catalogue number: 11-0451-85, eBioscience, San Diego, CA, USA), CD4 V500 (1:100, catalogue number: 560783, BD Biosciences, Franklin Lakes, NJ, USA), CD8 APC (1:400, clone: 56-6.7, catalogue number: MA110302, BD Biosciences, Franklin Lakes, NJ, USA), CD8a PB (1:100, catalogue number: 55816, BD Biosciences, Franklin Lakes, NJ, USA), CD62L PE (1:100, catalogue number: 104407, Biolegend, San Diego, CA, USA) and CD44 FITC (1:200, catalogue number: 553270, BD Biosciences, Franklin Lakes, NJ, USA). Intracellular staining was performed with GrzB PE (1:100, clone: NGZB, catalogue number: 12-8898-80, Invitrogen) and IFN-γ PE-Cy7 (1:100, clone: XMG1.2, catalogue number: 505826, Biolegend, San Diego, CA, USA).

**Measurement of cytokines**: Multiplex assays from mouse serum were performed using the Bio-Rad Bio-PlexTM 200 system (Bio-Rad, Hercules, CA, USA), following the Bio-Plex ProTM Mouse Cytokine Assay protocol. The cytokines IFN-γ, IL-2, IL-10, TNF-α and IL-12p40, were analyzed in mouse serum from C57BL/6N and *PKCθ*-KO mice on day 6 post-infection and compared to healthy animals.

**Statistics**: Statistical analysis and graph creation were both performed using GraphPad (San Diego, CA, USA, version 9.0). Results are depicted as box blots showing all points from min to max. Statistical differences between two groups were analyzed using a two-tailed unpaired Student’s *t*-test or two-way ANOVA. In the case of more than two datasets, data were analyzed by Kruskal–Wallis. Survival curves were assayed using the Log-rank Mantel–Cox Chi-squared test and linear regression curve. Significance was considered for *p* values below 0.05. Biological replicates (*n*) indicated in figure legends refer to the number of mice, and experimental replicates are displayed as *N*. Sample sizes were chosen based on historical data.

## 3. Results

***PKCθ*** **knockout provides protection against the cerebral manifestations of malaria and preserves BBB integrity in ECM.**

**Figure 1:**
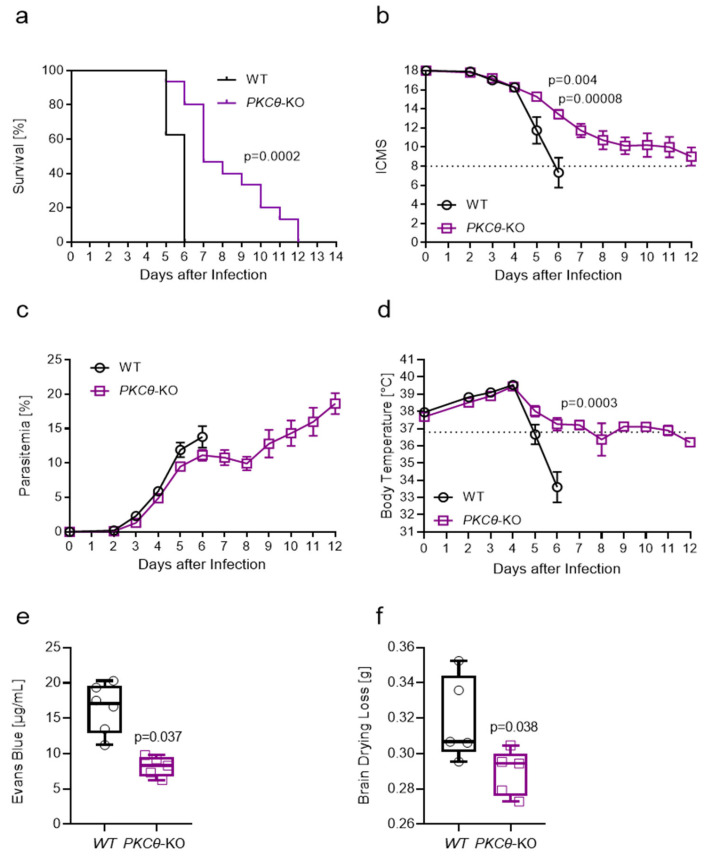
*PKCθ*-knockout protects from cerebral manifestations of malaria. (**a**) Survival curve of WT mice and *PKCθ*-KO mice infected with *PbA* (*n* = 8 WT vs. 15 KO; *N* = 3). Groups were compared with Log-rank (Mantel–Cox) test. (**b**) ICMS of *PbA* infected mice was assessed using various behavioral tests (two-way ANOVA, *n* = 8 WT vs. 15 KO; *N* = 3). (**c**) Parasitemia (percentage of parasitized erythrocytes) during infection was determined by daily blood smear analyses. (**d**) Body temperature was measured to depict typical fever curves of *PbA* infections (day 6: *t*-test, *n* = 8 WT vs. 15 KO; *N* = 3). (**e**) Evans Blue infiltration into brain tissue was assessed spectrophotometrically to quantify the integrity of the blood–brain barrier (day 6: *t*-test, *n* = 6 WT vs. 5 KO; *N* = 1). (**f**) Brain swelling is a typical hallmark of cerebral malaria. We used the drying loss of brains to determine the extent of water accumulation in encephalic tissue (day 6: *t*-test, *n* = 5; *N* = 1). The exact number of animals (n) per timepoint, censored animals before day 12 reaching euthanasia criteria andstatistical details for each timepoint are available in the deposited dataset [https://doi.org/10.5281/zenodo.17356230, accessed on 15 October 2025].

Using an experimental mouse model of CM based on *PbA* infection, we compared the susceptibility of *PKCθ*-deficient (*PKCθ*-KO) and wild-type (WT) C57BL/6N mice. *PKCθ*-KO mice showed a significant survival advantage following intravenous infection with 7.5 × 10^4^ *PbA* iRBCs (Figure 1a). Clinical scoring of neurological symptoms revealed that PKCθ-KO mice were largely resistant to severe ECM, consistently maintaining clinical scores above eight points (CM stage III), although ultimately, they succumbed to anemia caused by high parasite burdens 12–14 days post-infection (Figure 1b). There was no difference in parasitemia between genotypes (Figure 1c), indicating that the survival benefit was independent of parasite control and instead linked to protection from cerebral pathology. Body temperature monitoring showed a decline below 37 °C in WT mice with neurological symptoms, whereas *PKCθ*-KO mice maintained stable temperatures (around 37 °C) until late in the course of infection (Figure 1d). These findings confirm earlier results obtained with another *PKCθ*-knockout model and together identify PKCθ as a disease driver in ECM [11,12].

Protection from ECM in *PKCθ*-KO mice following *PbA* infection was associated with significantly less compromised BBB integrity. Intravenous Evans Blue injection revealed reduced dye extravasation into brain tissue in *PKCθ*-KO animals compared to WT controls (Figure 1e). Consistently, brain water content, quantified by weight loss after drying at 150 °C overnight, was significantly lower in *PKCθ*-KO mice (Figure 1f), indicating reduced cerebral edema and preservation of neurovascular integrity.

**Histological analysis revealed reduced hemorrhagic brain lesions in *PKCθ*-deficient mice.**

**Figure 2:**
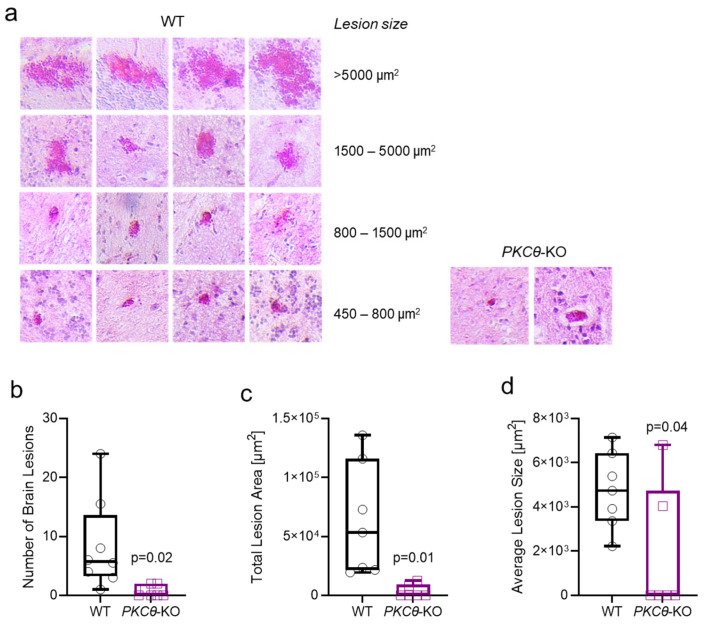
Loss of *PKCθ* preserves the brain against malaria-induced brain lesioning. (**a**) Representative images of brain sections stained with H&E. Representative sections with hemorrhagic lesions from WT and *PKCθ*-KO brains on day 6 p.i. with *PbA* are shown. (**b**) Quantification of the number of brain lesions per mouse brain (day 6: *t*-test, *n* = 8 WT vs. 7 KO; *N* = 2). (**c**) The area of hemorrhagic regions was determined using ImageJ (day 6: *t*-test, *n* = 7 WT vs. 6 KO; *N* = 2). (**d**) The average lesion size was calculated by dividing the number of brain lesions through the total lesion area (day 6: *t*-test, *n* = 7 WT vs. 6 KO; *N* = 2).

Moreover, when looking at the histological manifestations of *PbA*-infection—hallmarks of ECM—in *PKCθ*-KO and WT mice, it became apparent that hemorrhagic lesions in the brain were less prevalent and pronounced in the absence of *PKCθ* (Figure 2a) in H&E-stained brain sections at day 6 p.i. Both the number (Figure 2b) and size (Figure 2c,d) of lesions were markedly reduced in *PKCθ*-KO animals compared to WT mice. In WT animals, lesion size ranged from ~450 to >5000 µm^2^, whereas *PKCθ*-KO mice showed fewer and smaller lesions (450–800 µm^2^). Consequently, the total brain lesion area was substantially smaller in *PKCθ*-deficient mice (Figure 2c). Overall, these protective effects were accompanied by markedly less ECM-associated hemorrhagic tissue destruction, highlighting the neuroprotective impact of *PKCθ* deficiency during ECM pathology.

***PKCθ*-KO displayed less parasite sequestration and vessel obstruction.**

**Figure 3:**
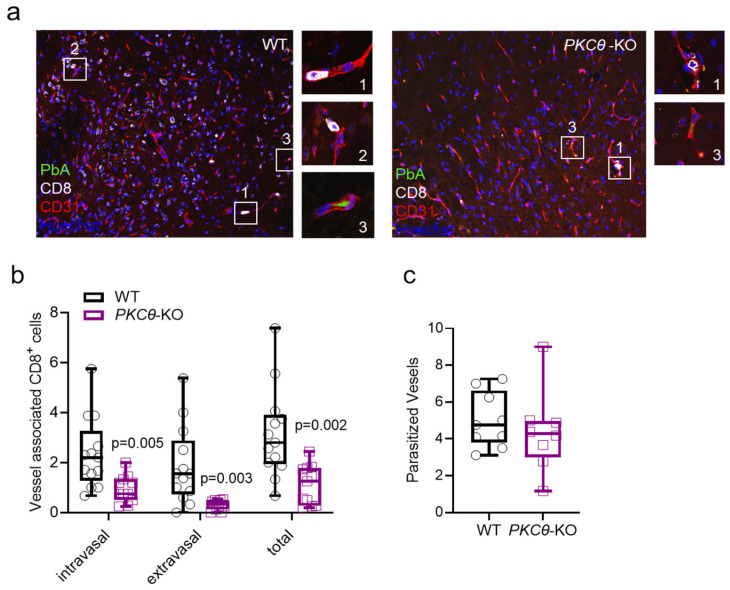
Representative images of immunofluorescence staining of brain sections. (**a**) WT and KO mice infected with *PbA* (day 6 p.i.), manifesting sequestration of GFP-expressing *PbA* parasites (green), as well as of CD8^+^ T cells (white) to the CD31^+^ ECs of the brain microvasculature (red; nuclei are illustrated in blue; 20×). (WT) Area (1) shows intravasal CD8^+^ cells, (2) accentuates extravasal CD8^+^ T cells and (3) illustrates a parasitized vessel in WT mice (40×). (KO) Area (1) shows intravasal CD8^+^ T cells, and (3) illustrates a parasitized vessel in KO mice (40×). (**b**) The amount of extravasal as well as intravasal and total CD8^+^ T cells per image is significantly lower in *PKCθ*-KO mice compared to WT controls. (**c**) There is no significant difference between the number of parasitized vessels between genotypes (WT *n* = 13, KO *n* = 11).

Consistently with the enhanced BBB integrity in *PKCθ*-deficient mice infected with *PbA*, we examined the sequestration of *PbA* parasites to ECs histologically using GFP-expressing *PbA*. This sequestration was comparable in WT and *PKCθ*-KO mice (Figure 3a,c). Contrary, and in line with the enhanced BBB integrity and fewer hemorrhagic lesions, fewer CD8^+^ T cells were found associated with vessels in the ECs of the brain microvasculature (CD31) in *PKCθ*-KO animals (Figure 3a,b). The sequestered lymphocytes were differentiated based on their location in relation to the brain microvasculature. The invasion of CD8^+^ T cells out of the blood vessels and into the brain tissue (extravasation) was substantially lower in *PKCθ*-KO mice than in WT controls. Similarly, the number of intravasal CD8^+^ brain-infiltrating lymphocytes (BILs) was approximately 60% lower in *PKCθ*-KO mice than in controls (Figure 3b). Overall, histological analysis of brains from infected *PKCθ*-KO mice revealed significantly fewer hemorrhages and less vessel obstruction compared to WT animals.

***PKCθ*-deficiency abrogates brain-infiltrating CD8^+^ T cell effector function during ECM.**

**Figure 4:**
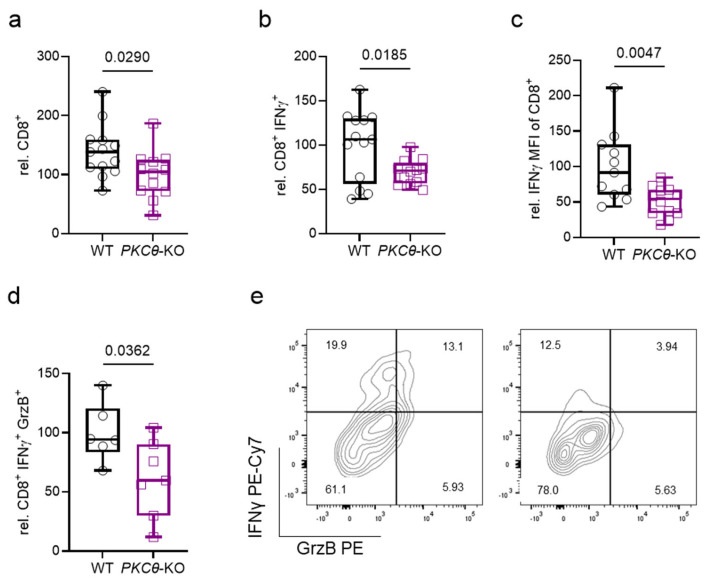
Flow cytometry analysis of BILs. (**a**) Fewer CD8^+^ T cells in *PbA*-infected *PKCθ*-KO brains compared to WT (results are depicted relative to WT). Cytokine FACS analysis after overnight TCR-re-stimulation of BILs (day 6: *t*-test, *n* = 13 WT vs. 12 KO; *N* = 3) revealed fewer IFN- γ producing CD8^+^ T cells (**b**), less IFN- γ mean fluorescence intensity (MFI) (**c**), and fewer IFN- γ/GrzB double-positive (**d**) CD8^+^ T cells from infected *PKCθ*-deficient animals relative to WT controls (results are depicted relative to WT; day 6: *t*-test, *n* = 6 WT vs. 7 KO; *N* = 2). (**e**) Representative FACS plots of CD8^+^ T cells producing IFN-γ and GrzB in the brain of WT and *PKCθ*-KO mice on day 6 p.i. (day 6: *t*-test, *n* = 13 WT vs. 12 KO; *N* = 3).

**Figure A2:**
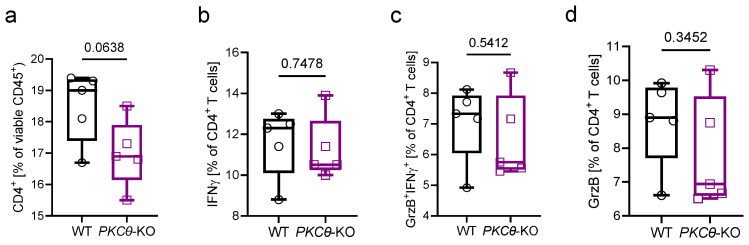
Flow cytometry analysis of BILs. (**a**) No difference in CD4^+^ T cells in PbA-infected PKCθ-KO brains compared to WT (results are depicted in % of viable CD45^+^ cells). Cytokine FACS analysis after overnight TCR-re-stimulation of BILs revealed no differences in IFN- γ (**b**), GrzB (**c**), nor IFN- γ/GrzB double-positive cells (**d**) in CD4^+^ BILs from infected PKCθ-deficient animals compared to WT controls (results are depicted in % of CD4+ cells; day 6: *t*-test, n = 5; *N* = 1).

Following the histological observation of fewer vessel-associated CD8^+^ T cells in brain sections of *PKCθ*-KO animals compared with WT controls, we performed flow-cytometric analysis of BILs and observed less CD8^+^ T cell abundance in brains of infected *PKCθ*-KO mice (Figure 4a). Further analysis revealed that, in addition to the marked reduction in BIL CD8^+^ T cell numbers, there was a profound loss of CD8^+^ T cell cytotoxic effector function manifested by reduced IFN-γ and GrzB production (Figure 4b–e). Off note, we could not observe differences in cytokine production in the brain-infiltrating CD4^+^ compartment (Figure A2a–d). These findings suggest that PKCθ directly regulates critical effector differentiation and, in turn, attenuates T cel-driven pathology and disease severity in ECM by suppressing the neurotoxic activity of cytotoxic CD8^+^ lymphocytes within the brain.

***PKCθ*** **inhibition antagonizes CD8^+^ T effector/memory differentiation in the spleen during ECM.**

**Figure 5:**
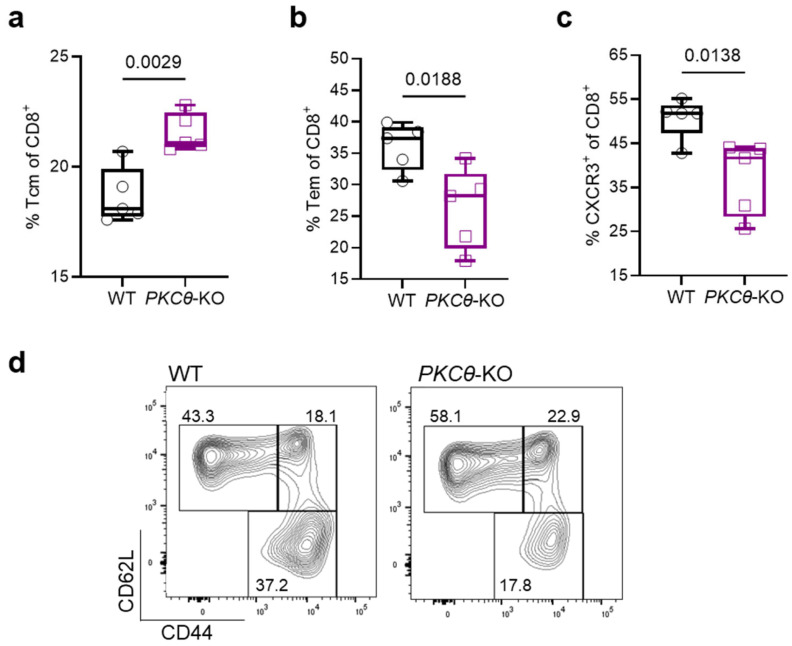
Flow-cytometric analysis of spleens from *PbA* infected mice. (**a**,**b**) FACS analysis of the differentiation status of splenic CD8^+^ T cell subsets (CD45^+^ CD3^+^ single lymphocytes) showed more central memory (T_cm_, CD62L^+^CD44^+^) and less effector memory (T_em_, CD62L^−^CD44^+^) in *PKCθ*-KO mice, as well as a more naïve like phenotype (CD62L^+^CD44^−^) (**d**). (**c**) Fewer CXCR3^+^CD8^+^ T cells in *PbA* infected brains from *PKCθ*-KO mice. (**d**) Representative FACS blots of the gating strategy for T_em_ and T_cm_. (day 6: *t*-test, *n* = 5; *N* = 1).

During ECM, when these PKCθ-mediated T cell differentiation processes are ineffective or absent, the dramatic reduction in neuroinflammation and protection from autoimmune-like fatal disease in *PKCθ*-deficient animal models can be explained. Consistent with the reduced hemorrhagic tissue damage in ECM brains lacking *PKCθ*, phenotypic characterization at day 6 post-infection revealed more central memory CD8^+^ T cells (Figure 5a,d) and fewer functional effector CD8^+^ T cells (Figure 5b,d). Notably, CD8^+^ BILs from *PKCθ*-KO mice displayed a more naïve phenotype; in contrast to the effector-memory phenotype predominant in WT animals.

Of note, CXCR3, a chemokine receptor typically present in effector memory CD8^+^ T cells, is markedly reduced in *PKCθ*-KO CD8^+^ T cells (Figure 5c), reflecting the shift away from the effector memory phenotype. This results in lowered responsiveness to CXCL9/CXCL10 signals and limits pathogenic CD8^+^ T cell trafficking to the brain. Consequently, *PKCθ* loss curtails neurovascular injury in experimental cerebral malaria by restraining the differentiation and subsequent migration of brain-infiltrating CD8^+^ T cells.

Taken together, these results indicate that reduced effector differentiation in CD8^+^ T cells of *PKCθ*-deficient mice likely underlies the suppression of cytotoxicity, thereby limiting endothelial cell injury, preserving BBB integrity, and ultimately improving survival in ECM.

***PKCθ*** **inhibition alters the levels of defined effector cytokines during ECM.**

**Figure 6:**
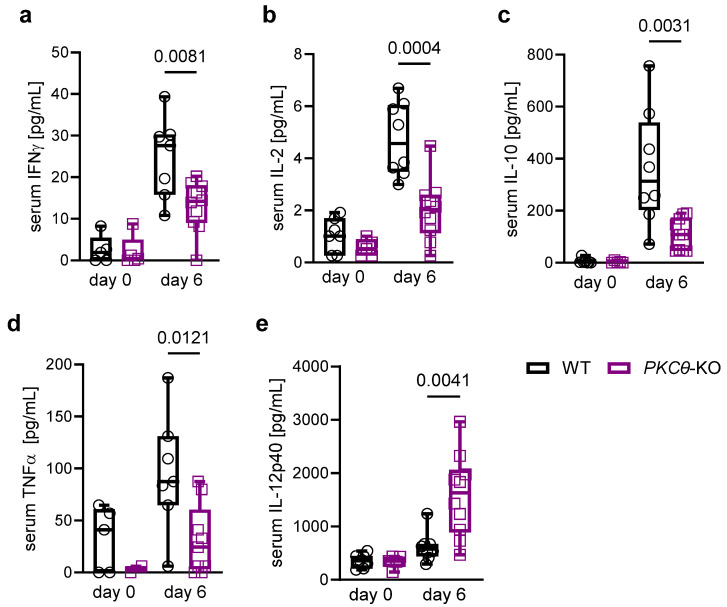
Knockout of *PKCθ* reduces systemic cytokine production by the adaptive immune system. (**a**–**e**) Serum cytokine levels were analyzed using Multiplex Immunoassays (WT vs. *PKCθ*-KO, day 6 p.i.; *t*-test, *n* = 5 for day 0 and *n* = 10–14 for day 6; *N* = 2). Mouse serum INF-γ level (pg/mL) (**a**), IL-2 level (**b**), IL-10 level (**c**), TNFα level (**d**), IL-12p40 level (**e**) on day 6 p.i. with *PbA*.

Genetic ablation of *PKCθ* significantly modulates the systemic immune response during ECM by reducing the levels of key pro-inflammatory cytokines in the bloodstream. Quantitative serum analysis revealed markedly lower concentrations of IFN-γ, interleukin-2 (IL-2), interleukin-10 (IL-10), and tumor necrosis factor-alpha (TNF-α), as well as higher concentrations of interleukin-12p40 (IL-12p40), in *PKCθ*-KO mice than in WT controls throughout the course of ECM (Figure 6a–e). This attenuation of systemic cytokine production is consistent with impaired effector differentiation of CD8^+^ T cells, which are central mediators of immunopathology in ECM. Consequently, reduced cytokine output is associated with reduced neuroinflammation and improved survival rates, further emphasizing the pivotal role of PKCθ in regulating pathogenic T cell functions during malaria-induced cerebral disease.

## 4. Discussion

Despite continuous and ongoing efforts to find new ways to combat CM, there is still no specific, highly efficacious adjunctive therapy for this disease. This is particularly necessary given that neuro-intensive care, which is essential for patients with CM, is widely unavailable in countries where severe cases of CM occur most frequently [16]. Therefore, there is an urgent and unmet medical need to identify novel druggable targets for human CM therapy [16]. Over recent years, several knockout mouse models have demonstrated survival benefits in *PbA*-induced ECM. Most of these models target genes involved in leukocyte migration to the brain and lungs, sequestration to the BBB, and modulation of T cell effector functions. For example, mice deficient in *PKCθ* [11], ApoE [17], Batf3 [18], ICAM1 [19], CXCL4 [20], CXCL10 [21], IFN-γR [22], Integrin αDβ2 [23], IL-12Rβ2 [24], and IL-4Rα [25] all exhibit reduced migration and sequestration of CD8^+^ T cells to the brain, often associated with lower expression of ICAM1, CD69 [11], CXCL9, and CXCL10 [26]. Nevertheless, absence of *PKCθ* [11] and IFN-γR [22] does not abolish lymphocyte infiltration to the lung. Beyond migration, cytotoxic functions of CD8^+^ T cells—such as Granzyme B, IFN-γ, and lymphotoxin-α secretion—are also compromised in mouse models deficient for Batf3 [18], IFNAR1 [26], IL-12Rβ2 [24], IL-4Rα [25], and ST2 [27], further supporting the concept that both trafficking and effector mechanisms are critical for ECM pathogenesis and protection.

There are critical differences between the two widely used *PKCθ* knockout mouse strains in ECM research. The Littman strain deletes exon 11, producing a truncated 365-amino-acid PKCθ fragment that may exert dominant-negative or off-target effects, potentially confounding phenotypic interpretations. In contrast, the Baier strain used here deletes exons 3 and 4, generating a true null allele with complete functional loss and eliminates residual or potentially confounding protein activity, providing a cleaner knockout system for dissecting PKCθ’s physiological roles. This distinction likely explains discrepancies between earlier reports and our findings, as the Baier model provides a more definitive system to probe PKCθ’s physiological roles in ECM. Our data strongly implicate PKCθ as a driver of CD8^+^ T cell-mediated neurovascular pathology, yet unresolved questions remain regarding CD8^+^ T cell-intrinsic versus additional effects.

Furthermore, while the genetic evidence supporting PKCθ as a therapeutic target is strong, direct translation to pharmacological intervention remains premature. Key challenges include achieving sufficient inhibitor potency, selectivity, and bioavailability, as well as ensuring effective CNS penetration for disease models like ECM. To accurately mimic the genetic ablation phenotype in vivo, future PKCθ inhibitors will require improved BBB penetration, longer half-lives, and optimized formulations or delivery methods. This is especially critical in light of pronounced differences in pharmacokinetics and drug metabolism between mice and humans, which impact both systemic exposure and translation of preclinical efficacy. Consequently, further studies employing next-generation, highly selective pharmacological tools are needed to definitively determine both causality and therapeutic potential.

Previous studies, including those from our laboratory, have demonstrated that PKCθ is activated downstream of the T cell receptor (TCR) complex and plays a pivotal role during the initial stages of T cell activation, driving proliferation and the production of essential cytokines such as IL-2, IFN-γ, and TNF-α [13]. Moreover, PKCθ is crucial in maintaining the delicate balance between activation and regulation of T cell responses, which underscores its significance in the pathogenesis of various autoimmune diseases, including autoimmune encephalitis, arthritis, and colitis [28,29,30]. Consistent with its essential role in promoting effector responses, the absence of PKCθ leads to a hyporesponsive T cell phenotype, characterized by reduced clonal expansion, impaired acquisition of an effector/memory phenotype, and a diminished capacity to mount robust CD8^+^ T cell-mediated immune responses.

Since CM is a largely immune-mediated disease, PKCθ has previously been proposed as a promising therapeutic target to downregulate brain inflammation by abrogating the exuberant CD8^+^ T cell-mediated cytotoxicity and subsequent BBB breakdown. Indeed, in the context of T cell-driven cerebral malaria pathogenesis, studies employing the ECM model have shown that targeting PKCθ mitigates immune-mediated pathology. Mechanistically, *PKCθ*-KO CD8^+^ T cells display defective proliferation and are impaired in their transition from the naïve (CD62L^+^CD44^−^) to the effector/memory (CD62L^−^CD44^+^) phenotype. These findings confirm PKCθ’s key role in T cell activation and effector differentiation and explain why *PKCθ*-KO mice are protected from ECM caused by pathogenic effector/memory CD8^+^ T cells.

Although accumulating evidence highlights the diverse roles of γδ T cells in malaria pathogenesis, the specific function of γδ T cells in *PKCθ*-KO mice remains incompletely characterized. *PKCθ*-deficiency is known to impair certain T cell signaling and effector functions, particularly in conventional αβ T cells, but its precise impact on γδ T cell activity requires further investigation. Notably, in our study, we found no differences in CD4^+^ T cell phenotype or cytokine production between experimental groups. Therefore, additional studies will be necessary to dissect the contributions of γδ T cells and to fully delineate the roles of other T cell subsets in cerebral malaria.

Thus, *PKCθ* blockade—as demonstrated by both previous studies and now confirmed using an additional genetic knockout strategy—prevents CM by halting the effector differentiation of pathogenic CD8^+^ T cells within the brain. This interruption in the pathogenic cascade leading to neurological damage and death positions PKCθ as a highly specific immunomodulatory target, uniquely effective against lethal neurological complications for which current antiparasitic treatments are insufficient. Collectively, these findings highlight PKCθ’s crucial role in mediating pathogenic T cell differentiation in ECM, suggesting that targeting PKCθ can suppress the activation-dependent effector functions of autoimmune-like brain-infiltrating CD8^+^ T cells, and represents a rational strategy to mitigate neuroinflammation and disease severity during ECM. This study confirms that *PKCθ* deficiency offers reliable protection against ECM, in line with previous findings. Both *PKCθ*-KO strains examined exhibited reduced systemic inflammation, fewer CD8^+^ T cells within the brain microvasculature, and preserved BBB integrity. These effects were associated with fewer hemorrhagic lesions, the prevention of ECM onset and improved survival.

Mechanistically, flow cytometry analysis revealed that protection was linked to reduced activation and cytotoxicity of CD8^+^ T cells in the brain, rather than altered parasite control, as parasitemia remained unchanged. These novel findings support a model in which loss of *PKCθ* skews T cell responses towards a less pathogenic effector differentiation profile, thereby limiting neurovascular injury. Despite limitations of the ECM model (e.g., species differences and absence of the liver stage), our data provide genetic evidence that CD8^+^ T cell-mediated neurovascular pathology in ECM is PKCθ-dependent. This positions PKCθ as a promising target for host-directed therapeutic interventions aimed at preventing or mitigating the symptoms of cerebral malaria.

Immune modulation represents a promising therapeutic avenue in cerebral malaria, with PKCθ emerging as a potential target due to its key role in T cell-mediated neurovascular pathology. Although genetic studies provide compelling evidence, translation into clinical therapies requires overcoming challenges related to drug specificity, bioavailability, and timing of intervention. Adjunctive treatments that attenuate harmful immune responses while preserving host defense are actively being pursued, and continued preclinical and clinical investigations will be essential for developing effective, targeted therapies that can complement antiparasitic drugs and improve patient outcomes.

## Data Availability

Raw values have been deposited and made publicly available. The dataset is accessible via ZENODO, under accession number [https://doi.org/10.5281/zenodo.17356230]. Further inquiries can be directed to the corresponding author.

## Abbreviations

The following abbreviations are used in this manuscript:

BBB	blood–brain barrier
BIL	brain-infiltrating lymphocyte
CD	cluster of differentiation
CM	cerebral malaria
EC	endothelial cell
ECM	experimental cerebral malaria
FCM	flow cytometry
H&E	Hematoxylin–Eosin
i.v.	intravenous
i.p.	intraperitoneal
ICMS	Innsbruck Cerebral Malaria Score
IF	immunofluorescence
iRBC	infected red blood cells
*PbA*	*Plasmodium berghei ANKA*
*Pf*	*Plasmodium falciparum*
p.i.	post-infection
PKCθ	Protein Kinase C-theta
RT	room temperature
SHIRPA	SmithKline Beecham, Harwell, Imperial College, Royal London Hospital, Phenotype Assessment
WT	wild-type

## Appendix A

**Table A1:** Innsbruck Cerebral Malaria Score (ICMS).

Parameter	Score	
**1: Body position**	Completely flat	0
	Lying on the side, Lying prone	1
	Sitting or standing	2
**2: Spontaneous activity**	None, resting	0
	Grooming, moderate to slow movement	1
	Vigorous, rapid movement, rearing	2
**3: Piloerection**	ruffled, with swaths of hair out of place	0
	dusty/piloerection	1
	Clean with sheen	2
**4: Startle response**	None	0
	Preyer reflex (backward flick of pinnae)	1
	Wince, jump	2
**5: Gait**	Incapacity	0
	Limited movement only, ataxic gait	1
	Normal	2
**6: Touch escape**	No response	0
	Mild to Moderate	1
	Vigorous (escape response to approach)	2
**7: Grip strength**	less than 50 Newtons	0
	50 to 100 Newtons	1
	more than 100 Newtons	2
**8: Body/Abdominal/Limb Tone**	Flaccid, no return of cavity to normal	0
	Reduced resistance	1
	Normal resistance	2
**9: Irritability**	None	0
	Struggle during supine restraint	1
**10: Vocalization**	None	0
	Provoked during handling	1
